# Editorial: Proteomics and Its Applications in Cancer

**DOI:** 10.3389/fonc.2021.772811

**Published:** 2021-10-07

**Authors:** Suman S. Thakur, Harsha Gowda, D. R. Mani, Bhaswati Chatterjee

**Affiliations:** ^1^ Proteomics and Signaling, Centre for Cellular and Molecular Biology, Hyderabad, India; ^2^ Queensland Institute of Medical Research (QIMR) Berghofer Medical Research Institute, The University of Queensland, Brisbane, QLD, Australia; ^3^ Computational Proteomics, The Broad Institute of Massachusetts Institute of Technology (MIT) and Harvard, Cambridge, MA, United States; ^4^ Chemical Science, National Institute of Pharmaceutical Education and Research, Hyderabad, India

**Keywords:** proteomics, cancer, post-translational modifications, mass spectrometry, quantitative proteomics, therapeutic, biomarkers

Proteomics has great potential to provide new insights into molecular and mechanistic processes that govern oncogenesis. Unraveling complex signaling mechanisms mediated by various post-translational modifications (PTMs), including phosphorylation, glycosylation, and ubiquitination, is an area where proteomics has the greatest impact. It is a valuable tool that could provide insight into the mode of action of new and established drugs. Mass spectrometry also allows the investigation of drug-protein interactions to enable the repurposing of drugs. High-throughput quantitative proteomics approaches including label-free and label-based quantification methods (SILAC, iTRAQ, and TMT) are extensively used to identify potential biomarkers and therapeutic targets. Here, we highlight some of these applications in studies published in this special issue.

## PTMs and Signaling

Post-translational modifications of proteins regulate the biological function of proteins and their role in signaling. Zhou et al detected hundreds of N-glycoproteins in airway fluid bronchoalveolar lavage (BAL) present in lung adenocarcinoma (ADC), squamous cell carcinoma (SQCC), and small cell lung carcinoma (SCLC). Further, a significantly elevated level of periostin was observed in all subtypes of lung cancer samples. Moreover, a lower dimethylation of asymmetric dimethylarginine (ADMA) containing protein was found in pancreatic ductal adenocarcinoma (PDAC) cell line PANC-1 (Wei et al). van Huizen et al reported a significantly lower degree of hydroxylation of collagen alpha-1 peptides in the tissue of colorectal cancer (CRC) and colorectal liver metastasis (CRLM) compared to colon tissues. Moreover, Suberoylanilide hydroxamic acid (SAHA) activates tumor suppressor p53 and Rb1 through phosphorylation and causes apoptosis in nasopharyngeal carcinoma (NPC) cells (Huang et al).

Interestingly, a significant increase of WNT10A, β-catenin, GSK-3ß, LMP7, and CacyBP/SIP were observed in renal cell carcinoma (RCC) compared to the control, suggesting the involvement of WNT/β-catenin pathway in RCC (Piotrowska et al). KIF21B was found to be highly upregulated in osteosarcoma, targeting PI3K/AKT pathway and playing an important role in the regulation of osteosarcoma cell proliferation and apoptosis (Ni et al).

## Diagnostic/Prognostic Biomarkers

Biomarkers can play an important role in the detection of early stage of cancer, recurrence, and treatment to reduce mortality rate. Tutanov et al reported SERPINA1, KRT6B, and SOCS3 as prognostic protein markers in breast cancer patients’ total blood exosomes. Proteins related to cholesterol synthesis were identified, including squalene monooxygenase (SQLE) and sterol-4-alpha-carboxylate 3-dehydrogenase, decarboxylating (NSDHL) in cytoplasmic lipid droplet proteomics of breast cancer cells (Zembroski et al). Further, neural precursor cells expressing developmentally downregulated protein 4 (NEDD4) play an important role in the maintenance of a significantly high number of characteristic cancer stem cells (CSCs) in triple-negative breast cancer (TNBC) (Jeon et al). Another study found the expression of Lactadherin or MFG-E8 in mammary epithelial cells is associated with intracellular deterioration (Verma et al). The junction plakoglobin (JUP) is an important biomarker for epithelial ovarian cancer and, together with Cancer Antigen 125 (CA125), may be used for screening ovarian cancer (Weiland et al). Kumar et al discussed blood cancer biomarker discovery and plasma proteomics using iTRAQ, TMT, MRM, and PRM. Zhu et al reported apoptosis genes BAK1 and CSE1L as potential diagnosis and prognosis markers for hepatocellular carcinoma.

## Therapeutics, Mode of Action, and Resistance

Wang et al studied the effect of lobaplatin in osteosarcoma cells with and without interleukin (IL)-6 using tandem mass tag (TMT) labeling and suggested upregulation of far upstream element-binding protein 1 (FUBP1), fragile X mental retardation syndrome-related protein 1 (hFXR1p), and rasGTPase-activating protein-binding protein 1 (G3BP1) and verified the result by PRM. They further found significantly high expression of FUBP1, hFXR1p, and G3BP1 in platinum-resistant osteosarcoma patients. Interestingly, more active ubiquitin-dependent protein catabolism and higher expression of WDHD1 were observed in the cisplatin-resistant strain A549/DDP as compared to A549 cells. Further, WDHD1 promotes MAPRE2 ubiquitination and causes cisplatin resistance in lung adenocarcinoma (Gong et al).

Gallegos et al carried out proteomics of CSC enriched with letrozole resistant breast cancer cell (LTLT-Ca) mammospheres and reported a significant increase of the chaperone protein midasin which is needed for maturation and nuclear export of the pre-60S ribosome. Nishimura et al attempted to find differences in cells with L858R and Ex19del mutations in epidermal growth factor receptor (EGFR) mutant lung adenocarcinomas to identify the efficacy of tyrosine kinase inhibitors.

## Insights Into Cancer Biology

The DNA replication licensing factor MCM2 was significantly expressed in lung squamous cell carcinoma patients (Pan et al). In endometrial carcinoma (EC), the F-box only protein 2 (FBXO2) was significantly upregulated and was related to its tumor stage. Further, the FBXO2 plays an important role as E3 ligase and causes ubiquitin-dependent degradation of Fibrillin1 (FBN1) (Che et al). The upregulation of myoferlin (MYOF), EGFR, and ephrin type-A receptor 2 (EPHA2) were reported in metastatic NPC cells using cell surface biotinylation and SILAC along with the interaction of MYOF with EGFR and EPHA2 (Li et al). Broto et al studied peripheral blood (PB) and bone marrow compartment (BM) samples of B-cell acute lymphocytic leukemia (B-ALL) and suggested the important role of transthyretin and interferon-gamma (IFN-γ) in B-ALL. Notably, the decrease of tumor necrosis factor-alpha (TNF-α) and interleukin 1 beta (IL-1β) was reported in a pesticide-exposed group (Pizzatti et al).

The secretome of Schwann cells (SCs), including matrix metalloproteinase-2, cathepsin D, plasminogen activator inhibitor-1, and galectin-1, may stimulate pancreatic cancer (PC) growth and invasion (Ferdoushi et al). Furthermore, bacterial proteomics of pancreatic head ductal adenocarcinoma (PDAC) suggested dysbiotic microenvironment with the occurrence of surfactin in PDAC and zeatin in gallstone (GS) patients (Arteta et al).

## Application of Proteomics Technologies

Multiple reaction monitoring was used to quantify peptides from Prostaglandin H2 D-Isomerase (PTGDS), Vitronectin (VTN), and Complement C3 (C3) using CSF from glioma and meningioma patients (Ghantasala et al). The transition of proteins related to meningioma pathobiology, including NEK9 and CKAP4, were observed in targeted proteomics using SRM assay (Mukherjee et al). Further, the changes in bone marrow interstitial fluid (BMIF) and serum proteome in multiple myeloma (MM) patients were reported using various proteomic methods, including SWATH-MS, suggesting the importance of haptoglobin, kininogen 1, and transferrin in MM (Chanukuppa et al). Vinaiphat et al discussed the role of different proteomics approaches in understanding the hypoxia-driven progression of cancer. Acland et al reported a proteomics study of premalignant lesions in endometrial intraepithelial carcinoma (EIC) and serous tubal intraepithelial carcinoma (STIC). Further, Gahoi et al studied cerebrospinal fluid (CSF) samples of low-grade glioma (LGG) and the glioblastoma multiforme (GBM) patient using protein microarrays.

Thus, this special issue highlights the application of proteomic technologies to gain deeper insight into cancer, with topics spanning biomarker discovery, pathogenesis, mechanistic details of signaling pathways, the effect of post-translational modifications, therapeutic agents and their mode of action, and computational proteomics.

## Author Contributions

ST, HG, DM, and BC conceived the idea and wrote the manuscript. All authors contributed to the article and approved the submitted version.

## Conflict of Interest

The authors declare that the research was conducted in the absence of any commercial or financial relationships that could be construed as a potential conflict of interest.

## Publisher’s Note

All claims expressed in this article are solely those of the authors and do not necessarily represent those of their affiliated organizations, or those of the publisher, the editors and the reviewers. Any product that may be evaluated in this article, or claim that may be made by its manufacturer, is not guaranteed or endorsed by the publisher.

